# Detecting Axial Ratio of Microwave Field with High Resolution Using NV Centers in Diamond

**DOI:** 10.3390/s19102347

**Published:** 2019-05-21

**Authors:** Cui-Hong Li, Deng-Feng Li, Yu Zheng, Fang-Wen Sun, A. M. Du, Ya-Song Ge

**Affiliations:** 1 Key Laboratory of Earth and Planetary Physics, Institute of Geology and Geophysics, Chinese Academy of Sciences, Beijing 100029, China; amdu@mail.iggcas.ac.cn (A.M.D.); ysge@mail.iggcas.ac.cn (Y.-S.G.); 2 University of Chinese Academy of Sciences, Beijing 100049, China; 3 CAS Key Lab of Quantum Information, University of Science and Technology of China, Hefei 230026, China; ldf2015@mail.ustc.edu.cn (D.-F.L.); bigz@mail.ustc.edu.cn (Y.Z.); fwsun@ustc.edu.cn (F.-W.S.)

**Keywords:** MW magnetic field, axial ratio, polarization, NV center

## Abstract

Polarization property characterization of the microwave (MW) field with high speed and resolution is vitally beneficial as the circularly-polarized MW field plays an important role in the development of quantum technologies and satellite communication technologies. In this work, we propose a scheme to detect the axial ratio of the MW field with optical diffraction limit resolution with a nitrogen vacancy (NV) center in diamond. Firstly, the idea of polarization selective detection of the MW magnetic field is carried out using a single NV center implanted in a type-IIa CVD diamond with a confocal microscope system achieving a sensitivity of 1.7 μT/$\sqrt{\mathrm{Hz}}$. Then, high speed wide-field characterization of the MW magnetic field at the submillimeter scale is realized by combining wide-field microscopy and ensemble NV centers inherent in a general CVD diamond. The precision axial ratio can be detected by measuring the magnitudes of two counter-rotating circularly-polarized MW magnetic fields. The wide-field detection of the axial ratio and strength parameters of microwave fields enables high speed testing of small-scale microwave devices.

## 1. Introduction

Building and measuring circularly-polarized microwave (MW) fields are of great importance in many fields like magnetic resonance [1,2] and mobile satellite communications [3,4,5]. Conventionally, precision measurement of MW field strength is realized by converting the field strength to easily-measurable electrical signals like using the calorimetric method [6] and the technique of peak demodulation [7]. The antenna axial ratio measurement is conducted with the help of a linearly-polarized antenna [4]. However, the calorimetric method is susceptible to environment temperature, and the technique of peak demodulation does not perform well at weak field strength. The assistant antenna perturbs the properties of the antenna under test (AUT) and usually has a large volume. Quantum-based MW field detection technologies, such as super quantum interference device [8] and cold atoms [9], which offer high sensitivity, require extreme measurement conditions. Vapor cell devices [10,11] that are capable of high sensitivity MW field sensing at near room temperature have a spatial resolution confined to sub-100 μm.

Recently, quantum sensing based on a point defect, the nitrogen vacancy (NV) center, in diamond has attracted wide attention for its long coherence time at room temperature [12]. It has already been deeply researched and applied to magnetic field, electric field, and temperature sensing [13,14,15] in many subjects. As its electron spin state can be coherently manipulated by the MW field, it can also be applied to MW field sensing. In previous works, the NV-based MW magnetic field sensing schemes mainly focused on detecting MW magnetic field strength [16,17].

In this paper, we propose a scheme to detect the axial ratio of an MW device with high resolution using NV centers in diamond. The axial ratio detection is based on MW magnetic field strength detection of the two counter-rotating circularly-polarized components of an MW field. An NV center is a negatively charged point defect in diamond consisting of a substitutional nitrogen atom and an adjacent carbon vacancy. Its ground electron-spin triplet state can be selectively and coherently manipulated by a specified circularly-polarized MW field. The MW magnetic field strength of specific polarization can be derived from Rabi frequency or magnetic resonance spectra contrast [16,18]. Firstly, the MW axial ratio detection is demonstrated with a single NV center in diamond with a conventional confocal microscope. Then, wide-field detecting of the MW magnetic field, which greatly promotes sensing speed, is conducted with a wide-field microscopy system. The MW axial ratio detection technique facilitates the test and design of MW devices aimed at quantum spin manipulation [19,20,21] and satellite communication.

## 2. Principle

**Figure 1:**
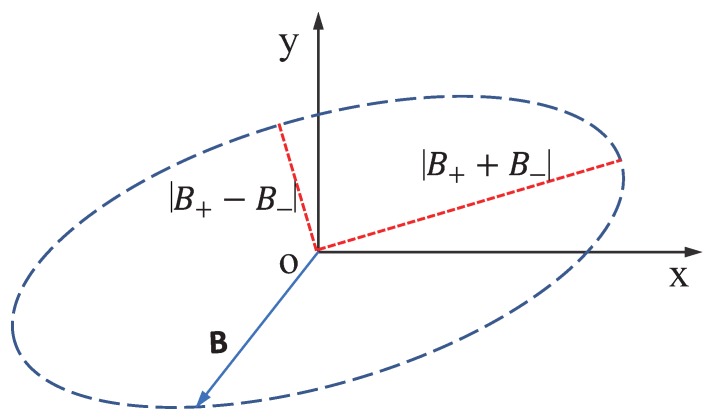
At a fixed point in space (or for fixed z), the magnetic vector B of a polarized microwave (MW) field traces out an ellipse in the xoy plane.

The axial ratio is an important parameter to describe a polarized MW field. Broadly, an arbitrarily-polarized MW field can be treated as an elliptically-polarized wave. The arbitrarily-polarized MW field spread along the z direction can be described as a combination of two circularly-polarized microwave fields, $B_{+}$ and $B_{-}$, spread along the z direction with the same frequency as the source MW field that rotate in different directions in the xoy plane (Figure 1).

(1) $$B=B_{+}+B_{-},$$

where $B_{+}$ ($B_{-}$) indicates the magnetic field strength of the $\sigma^{+}$ ($\sigma^{-}$) circularly-polarized field. The axial ratio can be described as the ratio of the magnitudes of the major and minor axes defined by the magnetic field vector. It can be figured out that the length of the major (minor) axis of the ellipse equals $2|B_{+}+B_{-}|$ ($2|B_{+}-B_{-}|$).

(2) $$AR=|\frac{B_{+}+B_{-}}{B_{+}-B_{-}}|.$$

Obviously, AR=1 for a circularly-polarized MW field, and AR=∞ for a linearly-polarized MW field.

**Figure 2:**
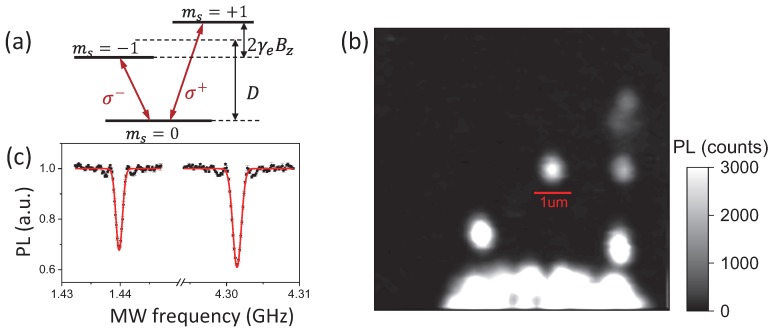
(**a**) The ground electron spin triplet state of the NV center with zero field splitting of D=2.87 GHz between the $m_{s}=0$ state and $m_{s}=\pm 1$ state. A magnetic field of $B_{z}$ along the direction of the NV axis leads to an energy level splitting of $2\gamma_{e}B_{z}$, where $\gamma_{e}=$ 2.8 MHz/G is the gyro-magnetic ratio of NV electron spin. The spin transition $m_{s}=0↔m_{s}=-1\left(+1\right)$ can be selectively addressed by the $\sigma^{-}\left(\sigma^{+}\right)$ polarized MW field. (**b**) Scanning picture of a diamond sample containing a single NV center. The marked bright spot is the single NV center used for MW field detection in this experiment. The other bright signals below are from ensemble NV centers. (**c**) Optically-detected magnetic resonance spectra of nitrogen vacancy (NV) center under a bias magnetic field of about 500 G. The data are fitted with the Gauss function. The first (second) dip at $f_{1}=1.44\;\mathrm{GHz}$ ($f_{2}=4.30\;\mathrm{GHz}$) corresponds to the transition $m_{s}=0↔m_{s}=-1\;\left(+1\right)$ state.

Axial ratio detection of MW fields with NV centers in diamond is possible as the strength of two counter-rotating MW magnetic fields can be selectively acquired by a spin-1 system. NV electron spin in its ground state can be optically pumped to the excited state and emit red fluorescence while decaying to the ground spin state.The spin state selective intersystem crossing (ISC) process [22] in the NV center enables the state read out and state initialization of NV electron spin. The NV electron in the $m_{s}=0$ state emits more photons then the NV center in the $m_{s}=\pm 1$ state. Successive illumination initializes the NV center to the $m_{s}=0$ state. As shown in Figure 2a, the electron spin ground triplet state of NV centers has an energy splitting of D=2.87 GHz between the $m_{s}=\pm 1$ state and $m_{s}=0$ state under zero magnetic field. The spin transition $m_{s}=0↔m_{s}=-1\left(+1\right)$, which can be simply treated as a two-level system (TLS) and can be selectively driven by $\sigma^{-}$($\sigma^{+}$) circularly-polarized microwave field perpendicular to the NV axis [20]. According to the semi-classical theory of light–matter interaction, the transition frequency, i.e., Rabi frequency $\Omega_{R}$, and the optically-detected signal contrast $C_{R}$ that corresponds to the electron spin state population of the TLS under a driving MW magnetic field can be written as:

(3) $$\Omega_{R}=\sqrt{{▵}^{2}+\Omega_{0}^{2}},\;\;C_{R}=C_{R0}\frac{\Omega_{0}^{2}}{\Omega_{0}^{2}+{▵}^{2}},$$

where $▵=\omega_{0}-\omega_{m}$ is the frequency difference between the frequency corresponding to the energy separation $\hbar \omega_{0}$ of the TLS and the frequency of the driving microwave field $\omega_{m}$. $\Omega_{0}=\gamma_{e}B_{mw}$ is the Rabi frequency at resonance, i.e., $\omega_{0}=\omega_{m}$; $\gamma_{e}$ = 2.8 MHz/G is the gyromagnetic ratio of NV electron spin. $B_{mw}$ is the amplitude of the resonant component of the driving microwave magnetic field. $C_{R0}$ is the maximum signal contrast between the $m_{s}=0$ state and $m_{s}=\pm 1$ state. Thus, the MW magnetic field strength of particular polarization can be deduced from Rabi frequency.

Furthermore, due to the Zeeman effect, the resonance frequency of transition $m_{s}=0↔m_{s}=\pm 1$ moves to $\omega_{\pm }=D_{gs}\pm \gamma_{e}B_{z}$ under a bias magnetic field $B_{z}$ along the NV axis (Figure 2a). This enables wide-band detecting of the microwave magnetic field. Simply by turning over the permanent magnet to apply bias magnetic fields in reversed direction, the strength of both $\sigma^{-}$ and $\sigma^{+}$ circularly-polarized components of the MW magnetic field at a certain frequency perpendicular to the NV axis that rotate in different directions can be extracted by detecting Rabi frequencies. Therefore, the axial ratio of the MW magnetic field at the location of the NV center perpendicular to the NV axis can be deduced.

## 3. Experiments and Results

### 3.1. MW Magnetic Field Detection Using a Single NV Center in Diamond

The detection of the MW magnetic field is firstly demonstrated with a single NV center in diamond. The experiment setup was based on a home-built confocal microscope [23]. A 532-nm green laser was used to initialize and detect the NV center. A microscope objective (NA = 0.9, Olympus, Kyoto, Japan) was used to focus the laser and collect fluorescence photons. The diamond sample was a single crystal synthetic (100)-oriented electronic-grade type-IIa diamond with dimension of $2\times 2\times 0.5\;{\mathrm{mm}}^{3}$ from the Element Six company. The diamond sample typically had less than a 0.03 ppb NV concentration before implantation. The investigated individual NV center was generated by ${}^{14}N$ ion implantation. The implantation dosage was $10^{11}\;{\mathrm{cm}}^{-2}$, and the estimated average depth of NV was about 20 nm. The MW field (R&S SMB 100A signal generator, Munich, Germany) was delivered to the sample via a coplanar waveguide (CPW) fabricated on the top surface of the diamond substrate. The diamond sample was mounted on a NanoCube Piezo System (PI 611.3S, Karlsruhe, Germany) enabling nano-scale scanning. A permanent magnet mounted on a three-axis translation stage applied a bias magnetic field of between zero and roughly 600 G, aligning with the NV axis (z axis). Figure 2b is a fluorescence picture obtained by scanning the diamond sample. The marked bright spot in the picture is a single NV center chosen for MW magnetic field sensing. Second order photon correlation measurement of the bright spot with strong antibunching observed at zero delay $g_{2}\left(0\right)∼0.11<0.5$ is proof that a single NV center was investigated [24] (data not shown).

**Figure 3:**
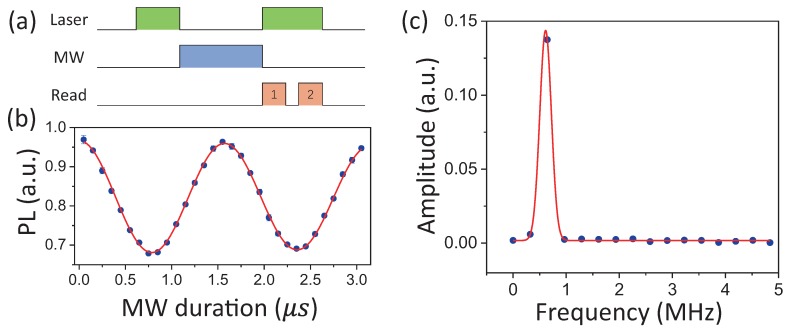
(**a**) Rabi frequency measurement sequence. A first green pulse of 3μs was applied to initialize the NV electron spin to the $m_{s}=0$ state; then, the MW field was applied to interact with the NV electron spin; finally, the electron spin state was determined by simultaneously applying the laser pulse and photon counts read pulse (0.3μs). To avoid the drift effect of the NV center on determining the NV state, the spin state photon counts were normalized to another photon counts read pulse that was applied in succession to the first one after the NV center was polarized again. (**b**) Rabi oscillation signal driven by a resonant MW field of 1.44 GHz. Each data point on the diagram was obtained by repeating the measurement sequences one million times. The oscillation frequency of 0.63 MHz indicates an MW magnetic field strength of $B_{\sigma^{-}}=0.22$ G at the NV spot. The fitting error yields a sensitivity of $1.7\;\mu T/\sqrt{\mathrm{Hz}}$. (**c**) Fourier transformation of (**b**).

To conduct polarization selective MW magnetic field sensing, a bias magnetic field was applied to split the resonant spectra lines corresponding to $m_{s}=0↔m_{s}=-1$ and $m_{s}=0↔m_{s}=+1$ transitions. In our experiment, a bias magnetic field of about 500 G was applied. As shown in Figure 2c, the optically-detected magnetic resonance (ODMR) spectra revealed an energy level splitting of 2.86 GHz between the $m_{s}=-1$ state and $m_{s}=+1$ state. Although the MW field from the signal generator was linearly polarized, the transition $m_{s}=0↔m_{s}=-1$ was only sensitive to the $\sigma_{-}$ MW field when the MW magnetic field strength was not too strong according to the rotating wave approximation in quantum optics [19]. We implemented the measurement of the strength of the $\sigma^{-}$ circularly-polarized component of the MW magnetic field with the NV center by detecting Rabi frequency. The MW field frequency was adjusted in resonance to the $m_{s}=0↔m_{s}=-1$ transition. The Rabi frequency detection sequence is shown in Figure 3a. A laser pulse was firstly applied to initialize the NV electron to the ground $m_{s}=0$ state. Then, an MW manipulation pulse was inserted. Finally, the population of the electron spin state was detected by another laser pulse. To obtain the manipulation frequency of the MW field, the MW pulse varied in time duration. Moreover, a photon count read pulse-2was applied after the NV center was polarized to the $m_{s}=0$ state again to provide a reference for drifts of the NV center. Figure 3b shows the Rabi oscillation detected with the MW field source power of about 7.8 mW applied. The oscillation signal was fitted by $1-C_{0}e^{-t/t_{0}}sin\left(wt+\phi \right)$. The manipulation frequency of 0.63 MHz indicated the $\sigma^{-}$ polarized MW magnetic field strength of $B_{-}=0.22$ G at the NV center spot perpendicular to the NV axis. According to the fitting error of $\delta B_{-}=0.0024$ G and detecting time of $t_{meas}=1000$ s of the Rabi nutation curve, the sensitivity for MW field detection was $\eta =\delta B\sqrt{t_{meas}}=1.7\;\mu T/\sqrt{\mathrm{Hz}}$. The Rabi oscillation frequency can also be derived by Fourier transformation (Figure 3c).

**Figure 4:**
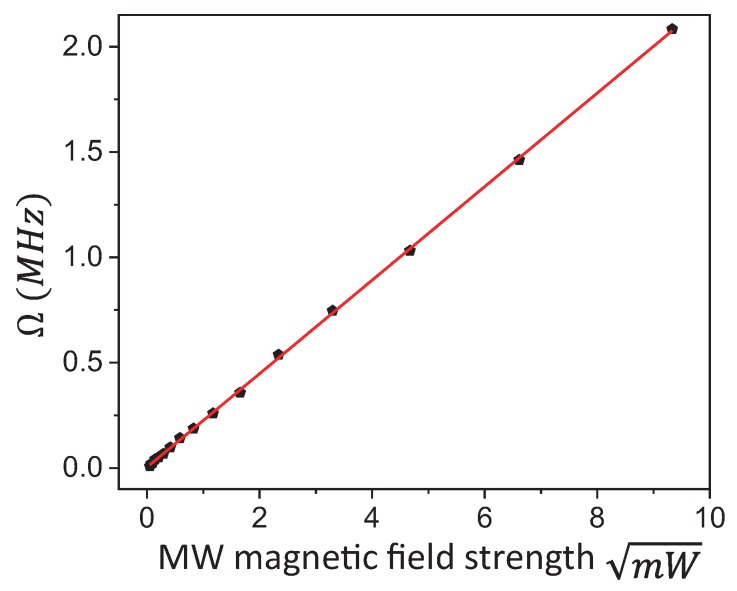
Rabi frequency versus MW source power applied. The data are fitted by a linear function.

As $B_{MW}\propto \sqrt{P}$, $\omega_{MW}=\gamma_{e}B_{MW}$, the Rabi frequency increased linearly with the square root of the resonant MW field power. The linear dependence was verified by detecting Rabi oscillation signals with different MW field powers applied. Figure 4 shows the linear dependence of Rabi frequency on microwave magnetic field strength. This is consistent with the measurement principle.

Turning over the permanent magnet to apply a bias magnetic field in the reverse direction, the $\sigma^{+}$ component of the MW magnetic field perpendicular to the NV axis can be detected similarly. Therefore, the axial ratio of the MW magnetic field at the NV point perpendicular to the NV axis can be derived.

### 3.2. Wide-Field MW Magnetic Field Imaging with Ensemble NV Centers

A single NV center is capable of detecting the strength of the particular circularly-polarized component of the MW magnetic field with high spatial resolution and high sensitivity. Combining the wide-field microscopy system and ensemble NV centers, the detection speed can be crucially improved, and the spatial resolution is only confined to the optical diffraction limit, which is generally at the micron or sub-micron degree. For many practical applications, wide-field microscopy that offers MW field strength imaging at a short time scale and requires simpler experimental technologies could be highly beneficial.

**Figure 5:**
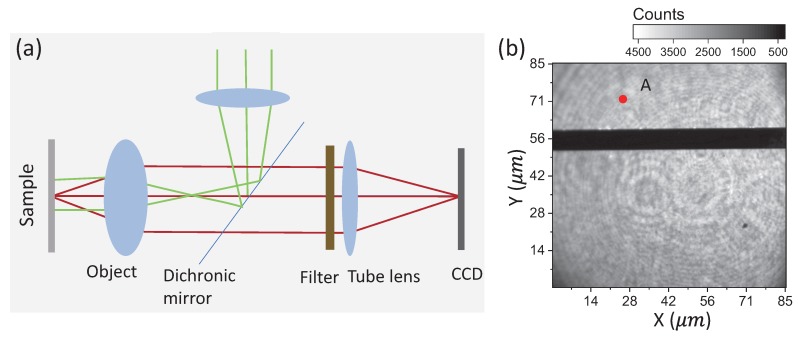
(**a**) Schematic diagram of wide-field fluorescence microscopy. The green light was focused at the back focal are of the object to excite NV centers with collimated light. The red fluorescence emitted by the NV centers was collected by the same object. The dichroic mirror was used to separate the laser and fluorescence. At last, the fluorescence was further filtered and focused on the EMCCD. (**b**) Wide-field fluorescence imaging of the diamond sample without the MW field fed in. The dark strip indicates the position of the metal MW strip line.

For wide-field detection of the MW magnetic field, the experiment was based on a home-built wide-field back focal imaging microscope system. As shown in Figure 5a, the green laser was focused at the back focal area of the object (NA=0.7) to illuminate the sample with collimated light. The red fluorescence emitted by ensemble NV centers at the focal plane was collected by the same objective and was separated from the source light by a dichroic mirror. Then, the light was filtered and focused on an EMCCD (Andor Ultra iXon 897, Oxford, UK) for imaging. The spatial resolution of the microscope system was about 700 nm, as the wavelength of fluorescence emitted from the NV center was roughly between 600 nm and 750 nm. The sample used in this experiment was a $2.6\times 2.6\times 0.3\;{\mathrm{mm}}^{3}$ general single crystal (100)-oriented CVD diamond from Element Six company with inherent ensemble NV centers. The inherent NV concentration in the diamond was estimated to be about $3\times 10^{14}\;{\mathrm{cm}}^{-3}$ (2 ppb) by comparing the fluorescence photon counts of the NV centers in the diamond to that of a single NV center in type-IIa diamond under the confocal microscope system. The fluorescence emitted from the diamond was ascribed to the NV${}^{-}$ center according to its zero photon line at 637 nm detected by the spectrometer FHR 640 (data not shown). A metal micro-strip line with a width of 8μm was fabricated on the top surface of the diamond to deliver the microwave field.

Figure 5b is a fluorescence picture taken by EMCCD with an exposure time of 2s. The metal strip line on the top surface blocked the fluorescence from the NV center, as shown in Figure 5b. Rough detection of the MW magnetic field was carried out by comparing the signals with and without the resonant MW magnetic field applied [18]. At a zero magnetic bias field, the NV ground electron spin energy splitting was $D_{gs}∼$ 2.87 GHz. Taking pictures of the same region as in Figure 5b with an MW field of f= 2.87 GHz applied, the NV fluorescence brightness decreased in the vicinity of the metal strip line (data not shown). The signal contrast decreased with the distance between NV centers and the MW strip line.

**Figure 6:**
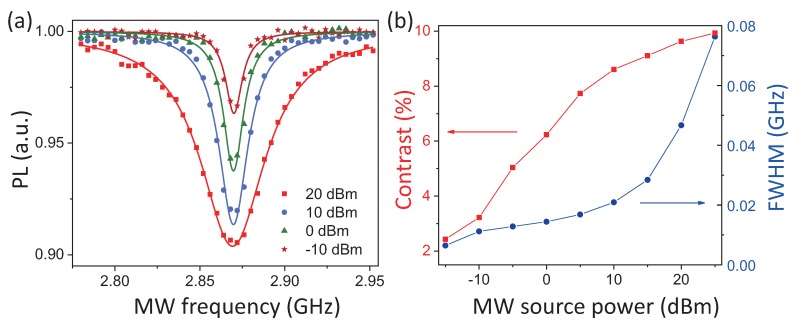
(**a**) ODMRspectra of Spot A in Figure 5b extracted with four different MW sources’ power fed in. The line width and dip contrast increase with the MW source power. (**b**) ODMR signal contrast (red square) and FWHM (blue dot) versus MW source power extracted from ODMR spectra of Spot A with different MW source powers fed in.

Taking pictures of the same region while scanning the MW frequency applied to the strip line, the ODMRsignal of the region can be extracted. To verify the wide-field MW magnetic field sensing method, ODMR signals of Spot A were extracted with the MW magnetic fields of different source powers P transmitted to the MW strip line (Figure 6a). Obviously, the magnetic resonance signal contrast increased with the power of the MW field applied, as the red square in Figure 6b indicates [18]. Thus, simply by tuning the NV electron spin transition frequency in resonance with the MW field, the qualitative MW magnetic field strength of specific polarization and spread direction [19] can be determined from the NV fluorescence signal contrast. Besides, the blue dot in Figure 6b shows that the full width at half maximum (FWHM) of the NV magnetic resonance signal decreased with reduced MW source power till a threshold value. The minimum FWHM of NV ODMR spectra reflects the NV electron spin decoherence property [18]. Moreover, the NV axial directions in the diamond were the same as the four tetrahedral diamond axes [25]. The MW magnetic field spread along each NV axis can be detected. Thus, the axial ratio of an MW field spread along an arbitrary direction can be extracted. Further applying the Rabi measurement sequence in the wide-field microscopy system, precise detection of MW magnetic field strength and the axial ratio is attainable [26,27].

## 4. Conclusions

In summary, we proposed a new method that can detect the axial ratio of microwave fields with high resolution for wide-field and a short time scale. The measurement method relied on detection of two circularly-polarized MW magnetic fields that rotate in different directions in the plane perpendicular to the NV axis. The axial ratio detecting of the MW magnetic field was firstly demonstrated using a single NV center in diamond, achieving a sensitivity of $1.7\;\mu T/\sqrt{\mathrm{Hz}}$. Then, wide-field detection of the MW magnetic field with an imaging field size of $85\times 85\;\mu m^{2}$ was displayed. The single NV center in the type-IIa CVD diamond investigated was generated by ion implantation, and the ensemble NV centers in the general CVD diamond were inherent. In a future study, we will apply this technique to test the polarization-controllable MW devices that we designed for selective manipulation of NV sublevels.The method we proposed can effectively benefit the design of MW fields with special polarization properties at the sub-millimeter to millimeter scale and thus benefit the development of techniques relying on quantum spin manipulation [28,29,30] and a small-sized antenna.
